# The *codA* gene as a negative selection marker in *Citrus*

**DOI:** 10.1186/s40064-015-1047-y

**Published:** 2015-06-17

**Authors:** Maria Luiza Peixoto de Oliveira, Ed Stover, James G Thomson

**Affiliations:** USDA-ARS Subtropical Insects and Horticulture Research Unit, Fort Pierce, FL 34945 USA; USDA-ARS Crop Improvement and Utilization Research Unit, Albany, CA 94710 USA

**Keywords:** *Citrus sinensis*, *codA* gene, Cytosine deaminase, Negative selection marker, 5-Fluorocytosine

## Abstract

The use of positive selectable marker genes is widespread in plant genetic transformation allowing transgenic cells to grow while repressing non-transgenic cells. Negative selectable markers, on the contrary, allow the repression or ablation of transgenic cells. The *codA* gene of *Escherichia coli* encodes cytosine deaminase that hydrolyzes 5-fluorocytosine (5-FC) into the cytotoxic compound 5 fluorouracil. We tested the transgenic expression of the bacterial *codA* gene in citrus as a conditional negative selection marker, with the goal of selecting against plant tissues in which a transgenic cassette has not been successfully removed. We developed transgenic citrus lines containing the selection cassette, *codA*::*nptII*, driven by double enhanced *CaMV35S* promoter, verified by Southern blot analysis, RT-PCR, *DsRed* expression and subjected these transgenic lines to a 5-FC sensitivity assay. We found that, while non-transgenic citrus were unaffected by the presence of 5-FC, all of the transformed lines displayed symptoms of toxicity, indicating that the *codA* gene could be used as a negative selectable marker in Citrus, for post-transformation detection of the removal of undesired sequences.

## Background

The expression of a negative selectable marker gene causes either immediate or conditional cell death in transformed cells and therefore allows for the selection of cells lacking these marker genes (Koprek et al. 1999).

In several plant species, the *codA* gene from *Escherichia coli*, encoding cytosine deaminase, has been successfully used as a negative selection marker. The usefulness of *codA* as a conditional toxic gene has been explored in various *Agrobacterium*-mediated transformation protocols (Babwah and Waddell 2000; Dutt et al. 2008; Koprek et al. 1999; Rommens et al. 2004; Schaart et al. 2004). The gene product of *codA* deaminates cytosine to uracil, and also converts the uracil analog 5-fluorocytosine (5-FC) into 5-fluorouracil (5-FU). This substance is metabolized to 5-fluorouridine 5%-triphosphate and 5-fluoro-2%-deoxyuridine 5%-monophosphate, inhibiting both RNA and DNA synthesis, resulting in cell death (Andersen et al. 1989). Therefore, 5-FC is toxic for cells of several plant species transformed with the *codA* gene (Gallego et al. 1999; Kobayashi et al. 1995; Koprek et al. 1999; Perera et al. 1993). In this work, the *codA* coding sequence was isolated from the *E. coli* genome and cloned as a fusion to the *nptII* gene in the pCTAGV-KCN3 binary vector under control of the double enhance *CaMV35S* promoter. *Agrobacterium*-mediated transformation of citrus was carried out in order to evaluate the efficiency of selection using the *codA* gene as a negative marker. Non-transgenic and *codA*-transgenic internode segments were cultivated on selective medium containing 5-FC, showing that 5-FC did not affect the growth of non-transgenic citrus cells, whereas it inhibited shoot regeneration from *codA*-expressing transgenic cells. This positive–negative selectable marker may be applied to enhance recombinase-mediated cassette exchange for site-specific chromosomal integration in *citrus* (Wang et al. 2011).

## Methods

### Plant material

*In vitro*-*grown* etiolated epicotyls segments of ‘Carrizo’ citrange (*Citrus sinensis* × *Poncirus trifoliata*) and ‘Hamlin’ sweet orange [*Citrus sinensis* (L.) Osbeck.] were used as source of explants. Surface-sterilized seeds were inoculated in solution containing 12 ml of hormone-free half-strength MS basal medium (Murashige and Skoog 1962), supplemented with 3% sucrose and solidified with 8.0 g l^−1^ agar (Sigma-Aldrich) for germination. The test tubes were capped with polypropylene closures. The cultures were incubated in a growth chamber at an average temperature of at 27 ± 1°C under dark conditions for 6 weeks. The epicotyl portions of etiolated seedlings were cut transversally into 0.8–1 cm segments and used for transformation experiments.

### Bacterial strain and vector

**Figure 1:**
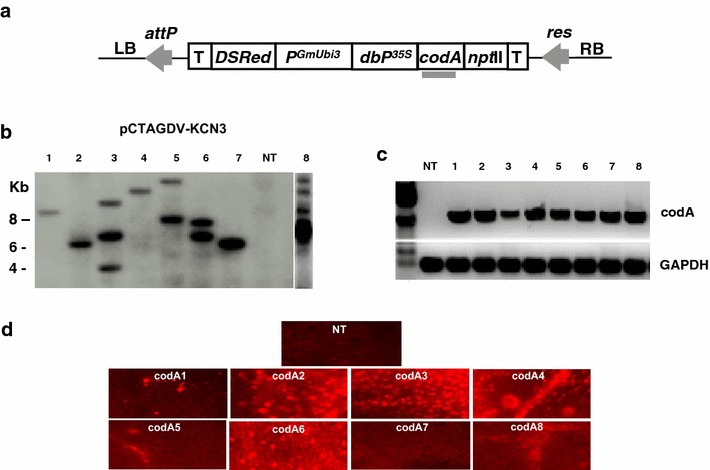
Schematic representation of the binary vector construct used and molecular analysis of representative transgenic lines. **a** Schematic representation of the *Agrobacterium* binary vector T-DNA carrying the *codA* gene. The binary vector pCTAGV-KCN3 is a pCambia 390 derivative harboring the *codA*:*nptII* fusion gene under the control of double enhanced CaMV 35S promoter (*dbP35S*) and nopaline synthase terminator, while the *DsRed* gene is expressed by the soybean ubiquitin3 promoter (*P* ^*GmUbi3*^) and 35S terminator; *codA* probe *gray bar.* The *attP* and *res* are recombinase recognition sites for the Bxb1 and CinH recombinase enzymes, respectively. **b** Southern blot analysis of non-transgenic and transgenic *codA* containing citrus lines. Lanes #1–8 are codA transgenic lines, NT is non-transgenic Carrizo; **c** RT-PCR analysis of non-transgenic and transgenic codA lines*. NT* non-transgenic Carrizo, *1–8* codA transgenic lines (same lines sequence number as shown in **b**); **d** Fluorescence microscopy of leaves derived from pCTAGV-KCN3 containing transgenic plants. Expression of the *DsRed* fluorescent marker gene in the non-transgenic and transgenic shoots under the Olympus Provis AX70 fluorescent microscope at ×10 magnification using DsRed-specific filters.

Plasmid pCTAGDV-KCN3 (Figure 1a) was transformed into *A. tumefaciens* EHA 105 (Hood et al. 1986) using freeze–thaw method (Höfgen and Willmitzer 1988). The plasmid pCTAGDV-KCN3, based on the pCambia390 vector backbone, contains the double enhanced 35S promoter constitutively expressing fusion gene *codA*::*nptII* a bifunctional selectable marker, and a *DSRed* visual reporter gene constitutively expressed by the *GmUbi3* promoter (Hernandez-Garcia et al. 2010). The pCTAGDV-KCN3 binary vector is a new plant genetic transformation vector contains a recombinase recognition site platform that enables recombinase mediated cassette exchange (RMCE) to target gene(s) of interest and remove unneeded DNA sequences, such as antibiotic resistance genes (Wang et al. 2011).

### Plant transformation and regeneration of transgenic plants

Epicotyl tissue of Carrizo citrange was inoculated for 15 min with an overnight culture of bacteria diluted to OD 600 = 0.4 and blotted on sterilized filter paper to remove excess bacteria in suspension. The infected epicotyl tissue was co-cultivated for 3 days on MS basal medium containing 3% sucrose, 1 mg l^−1^ BAP, 100 mg l^−1^ acetosyringone, 8.0 g l^−1^ agar and incubated at an average temperature of 24 ± 1°C in the dark. After 3 days of co-cultivation, the explants were transferred to MS medium containing 300 mg l^−1^ timentin (ticarcillin and clavulanate), 250 mg l^−1^ cefotaxime, and 100 mg l^−1^ kanamycin. After 45 days in culture on kanamycin selection media, putatively transformed shoots (3–5 cm in height) were transferred to root-induction medium (RIM), consisting of ¼ MS strength medium, 2% sucrose, 0.25% Gelrite, 2.5 mg l^−1^ IBA, 0.5 mg l^−1^ NAA, and 0.0025 mg l^−1^ spermidine. After rooting, plants with developed root systems were transferred to sterile soil cones, covered with plastic bags and gradually exposed to ambient humidity in a growth chamber, over a period of 15 days. Acclimated plants were transferred to greenhouse for maturation.

### Molecular analyses

The genomic DNA was isolated from leaves of transgenic and non-transgenic plants, as previously described (Štorchová et al. 2000). The concentration of DNA was measured using a Nanodrop 2000c (Thermo Scientific). For each sample, 100 ng of genomic DNA in 25 µl volume was used in a PCR with the primers codAORF70F60 and codAORF1137R60 primers (5′-CAAGACCCTTCCTCTATATAAG-3′ and 5′-CGAGTTCATAGAGATAACCTTC-3′, respectively) to detect *codA* expected fragment size 1,067 bp. As a control, DNA from non-transformed cv. Carrizo was amplified.

### Southern blot

For Southern-blot analysis, 5 µg of genomic DNA of non-transgenic and transgenic plants were digested with *Eco*RI for 6 h at 37°C and separated by electrophoresis on a 0.8% (w/v) agarose gel. The DNA was then transferred to a Hybond-N membrane (Amersham) and hybridized with the ^32^P-labelled *codA* sequence (produced by Promega TaqTM polymerase using primers codAORF70F60 and codAORF1137R60).

### RT-PCR

Total RNA was isolated from leaves of non-transgenic and transgenic Carrizo citrange plants, using RNeasy Plant Mini Kit (Qiagen). RNA was digested with DNase I (Ambion) and the cDNA was synthesized using SuperScript Synthesis Kit (Invitrogen). Semiquantitative PCR was performed, using standard amplification mixtures containing 1.5 mM MgCl_2_, 200 μM each dNTP, 2.0 μl of cDNA, 2 U *Taq* DNA polymerase, and 0.25 μM *GAPDH* primers (5′-GGAAGGTCAAGATCGGAA-3′ and 3′-TCAA CGTCCCTCTGCAAGATGACTCT-5′) or *codA* primers (5′-CAAGACCCTTCCTCTATATAAG-3′ and 5′-CGAGTTCATAGAGATAACCTTC-3′) in a 25 μl final volume. Amplification reactions were conducted in the following conditions: 1 cycle at 94°C for 4 min, followed by 25 cycles of 1 min denaturation at 94°C for 1 min, 1 min annealing at 60°C for the *codA* gene or 55°C for the citrus *GAPDH* gene (endogenous control), and 1 min elongation at 72°C. Finally, PCR amplified products were separated on a 1.2% agarose gel and visualized by ethidium bromide staining.

### Expression of DSRed

Expression of Red fluorescent marker genes in the transgenic and non-transgenic shoots were examined via Olympus Provis AX70 fluorescent microscope (Olympus) at 10× magnification and the appropriate filter for detection of the red fluorescence of the *DSRed* gene, which has an excitation maximum at 545 nm and emission maximum at 600 nm. The pictures were taken using a Q Color 5 Olympus camera with Q Capture software.

### 5-FC sensitivity assay

Transgenic lines confirmed by PCR, Southern blot, and RT-PCR were propagated and 18-month-old internodes were then cultivated in SIM supplemented with Kanamycin (Kan) and different concentration of 5-FC, in order to study both positive and negative selection phenotypes. Internode segments of 0.8–1 cm length from transgenic and non-transgenic plants were surface sterilized (70% ethyl alcohol for 120 s followed by 25% bleach for 15 min and washed three times with water), placed in Petri dishes 120 × 20 mm containing 25 ml of shooting induction media (SIM) based on the nutrients and vitamins of WPM medium (Lloyd and McCown 1980) supplemented with 2 mg l^−1^ BAP with five different concentrations of 5-FC (Sigma) (0, 50, 75, 100, 200, or 400 mg l^−1^), kept in the dark for 3 weeks at 26°C after which they were transferred to 16/8 h light/dark cycles at 27 ± 1°C. Non-transgenic plants were also tested for their response to various 5-FU (Sigma) concentrations (10, 20, 40, 80 and 100 mg l^−1^). 5-FC and 5-FU were dissolved in Milli-Q water at 65°C at concentration of 10 g l^−1^. These solutions were filter sterilized and immediately added to SIM medium at ~65°C to indicated dilutions. Counts of percentage of regeneration and numbers of shoots per explants were taken after 70 days. Results were expressed as the mean and compared using the Tukey’s multiple comparison test at 5% probability for 5-FC variables studied.

### Rooting assay using selective and non-selective media with 5-FC

Adventitious shoots of non-transgenic and transgenic line *codA2* were grown in SIM, and then transferred to root-induction medium (RIM), as previously described, containing 0, 50, 75, 100, 200 or 400 mg l^−1^ FC. Frequency of rooting was scored after 45 days in RIM.

## Results

### Selection and regeneration of transgenic plants

We have designed a transformation construct based on the pCambia390 backbone binary vector. The T-DNA contains a neomycin phosphotransferase II gene (*nptII*), which confers kanamycin resistance in plants as a positive selectable marker and the cytosine deaminase gene (*codA*) as the conditional negative selectable marker. The *codA* and *nptII* coding sequences are translationally fused as a single cassette and expressed by the ds35S promoter and terminator. The *DSRed* gene was included as a viable visible marker for early transformation detection (Figure 1a).

A total of 335 transgenic citrus lines from Carrizo and Hamlin were generated after 8–10 weeks of culturing epicotyl explants on regeneration/selection medium with kanamycin (100 mg l^−1^). These shoots grew normally and rooted.

### Molecular characterization of the regenerated plants

Putative transgenic plant lines were analyzed using the *codA* specific primer set to determine the presence of the negative marker (not shown). T-DNA copy number was determined using Southern blot analysis of an *EcoRI*-digested genome from 123 transgenic lines in Carrizo and 15 in Hamlin using a P^32^-labelled *codA* sequence as a probe. No signal was detected in the DNA from non-transgenic plants included as a negative control. Since there is only one *EcoRI* site in the transformed vector a second *EcoRI* site must be derived from the citrus genomic DNA and diagnostic for the number of T-DNA cassettes present. The number of T-DNA cassettes varied from one to five with most of the lines tested having one or two copies (examples in Figure 1b). Sixty-five plants from Carrizo and nine from Hamlin showed a single copy T-DNA integration site. Transgenic plants with single insertions were selected for further research.

Four lines with a single copy T-DNA insert (codA lines# 1, 2, 4, and 7) and four with multiple copy inserts (codA lines# 3, 5, 6, and 8) were analyzed by Reverse-Transcriptase-PCR (RT-PCR). The results of RT-PCR using *codA* specific primers showed that the transgene is transcribed in the transgenic plants (Figure 1c). Both transgenic and non-transgenic plants were examined by fluorescent microscope. Red fluorescence resulting from production of the DSRed protein was clearly visible in the transgenic lines. While the red fluorescent signal strength varied between lines, all of the transformed lines exhibited activity, while fluorescence was absent in non-transgenic citrus (Figure 1d).

### Negative selection assay

To determine whether the 5-FC CodA metabolic product, 5-FU, was an effective toxic agent on citrus internode segments, non-transgenic tissue was placed on media containing different concentrations of 5-FU (Table 1). A concentration of 20 mg l^−1^ resulted in strong reduction of the percentage of regeneration as well as number of shoots per explants in non-transgenic internode tissue. The resulting shoots showed abnormal leaf development and severe reduction in shoot length. At concentrations of 80 and 100 mg l^−1^ there was no detectable regeneration.

**Table 1:** Negative selection determination with 5-fluorouracil

Plant	5-FU Selection (mg l^−1^)											
	0		10		20		40		80		100	
	Regeneration (%)^1^	Shoots/explant^2^	Regeneration (%)^1^	Shoots/explant^2^	Regeneration (%)^1^	Shoots/explant^2^	Regeneration (%)^1^	Shoots/explant^2^	Regeneration (%)^1^	Shoots/explant^2^	Regeneration (%)^1^	Shoots/explant^2^
NT	71 ± 9.64	2.5 ± 0.6	67 ± 10.26	1.8 ± 0.79	8 ± 3.60	0.3 ± 0.2	2.6 ± 2.51	0.07 ± 0.06	0	0	0	0

Regeneration frequency and number of shoots per internode explant of non-transgenic (NT) citrus in regeneration medium supplemented with various concentrations of the toxic metabolite 5-fluorouracil (5-FU). Regeneration frequency and number of shoots per explants were taken after 70 days.

Data are the means of three independent experiments. Values are regeneration % (1) and number of shoots per explants (2) ±standard deviation between repetitions.

The effects of negative selection were examined on three transgenic Carrizo lines with one, two or three copies of the transgene cassette (codA lines# 2, 3, 6, respectively; Figure 1c), as well as on Carrizo non-transgenic. Eighteen month old internode tissue from the transgenic and non-transgenic lines were subjected to five levels of 5-FC. At 50 and 75 mg l^−1^ of 5-FC significantly affected shoot growth of *codA* expressing internode tissue in all three transgenic lines tested, causing the percentage of regeneration and number of shoots per explants to decrease by about 50 and 75%, respectively (Table 2). Non-transgenic internode tissue regenerated and produced a normal number of green and healthy shoots per explant at these concentrations.

**Table 2:** Negative selection determination with 5-fluorocytosine

5-FC selection (mg l^−1^)	Plant							
	NT		codA2		codA3		codA6	
	Regeneration (%)^1^	Shoots/explant^2^	Regeneration (%)^1^	Shoots/explan^2^	Regeneration (%)^1^	Shoots/explant^2^	Regeneration (%)^1^	Shoots/explant^2^
0	80 ± 4.93^a^	2.9 ± 0.52^a^	84 ± 6.08^a^	3.4 ± 0.65^a^	68 ± 12.50^a^	2.9 ± 0.32^a^	71 ± 11.71^a^	2.2 ± 0.40^a^
50	75 ± 8.02^a^	3.1 ± 0.52^a^	44 ± 10.39^b^	1.1 ± 0.49^b^	31 ± 10.01^b^	0.8 ± 0.40^b^	40 ± 13.74^b^	1.13 ± 0.25^b^
75	78 ± 2.08^a^	2.7 ± 0.40^a^	14 ± 7.50^c^	0.8 ± 0.35^b,c^	21 ± 9.16^b^	0.7 ± 0.21^b,c^	24 ± 11.53^b^	0.7 ± 0.28^b^
100	73 ± 6.11^a^	2.1 ± 1.10^a,b^	0^c^	0^c^	0^c^	0^b,c^	0^c^	0^c^
200	64 ± 6.05^a^	1.9 ± 0.46^a,b^	0^c^	0^c^	0^c^	0^b,c^	0^c^	0^c^
400	10 ± 7.09^b^	0.9 ± 0.7^b^	0^c^	0^c^	0^c^	0^b,c^	0^c^	0^c^

Internodes from non-transgenic and three transgenic lines, carrying the *codA::nptII* gene, were placed in regeneration medium supplemented with various concentrations of 5-FC. Regeneration frequency and number of shoots per explants were taken after 70 days.

Data are the means of three independent experiments. Values are regeneration % (1) and number of shoots per explants (2) ±standard deviation between repetitions. Values in a column followed by a common letter are not significantly different according to Tukey’s multiple range test (P < 0.05). Lines codA2, codA3, and codA6 have one, three, and two transgene cassette copies, respectively.

**Figure 2:**
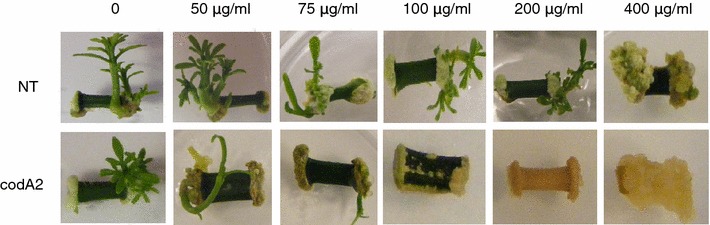
Phenotypes of non-transgenic and *codA* transgenic line *Carrizo citrange* internode segments on 5-FC containing media. Eighteen-month-old internodes of the codA2 transgenic line and non-transgenic Carrizo control (NT) were subjected to five levels of 5-fluorocytosine (5-FC). Internodes segments were placed on a Petri dish with shoot-induction medium containing various 5-FC concentrations. The explants were grown for 21 days under dark condition at 26°C and then transferred to light/dark condition at 27 ± 1°C.

At 100 mg l^−1^ of 5-FC, non-transgenic internode tissue was again similar to untreated plant material, while the transgenic internode tissue completely failed to regeneration shoots. However, growth and development of non-transgenic tissue was inhibited at 400 mg l^−1^ 5-FC, giving an upper limit to the use of this compound as a selective agent with citrus. Figure 2 summarizes results observed using the different levels of 5-FC on shoot growth of *codA* expression transgenic internode tissue compared to non-transgenic.

**Figure 3:**
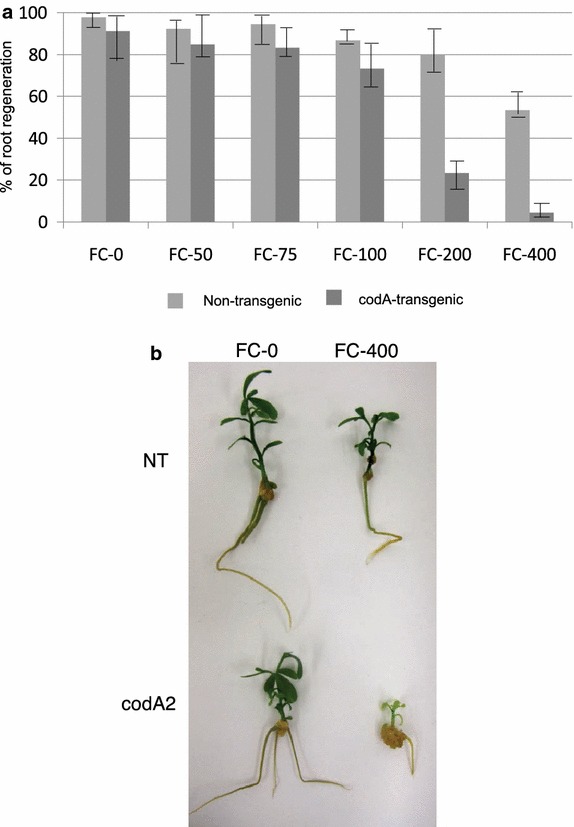
Rooting of regenerated shoots from non-transgenic and *codA* transgenic internodes as a function of 5-FC selection. **a** Root regeneration was evaluated 45 days after the transfer of the shoots to rooting media. The *bar chart* shows the average frequency of root regeneration and the standard error of means for root induction using three independent experiments with a total of 30 shoots per treatment of non-transgenic and the codA2 transgenic line. The *Y axis* represents the average percentage of explants showing root growth. *X axis* reports the concentrations of 5-FC. **b** Demonstration of effects of 0 and 400 mg l^−1^ 5-FC on Carrizo non-transgenic and transgenic line codA2 during rooting induction. Adventitious shoot from non-transgenic and codA2 transgenic line when placed on root-induction medium supplemented with various concentrations of 5-FC, as indicated in (**a**), after 45 days.

In order to test rooting induction in the presence of 5-FC, shoots from both non-transgenic and codA transgenic line #2 (codA2) were placed on rooting induction media (RIM) containing various concentrations of 5-FC, as indicated in Figure 3a. At 400 mg l^−1^ of 5-FC in RIM, few shoots from the transgenic line produced roots (4.4%), and when roots were produced they were small and stunted, and leaves appeared yellow (chlorotic) when compared to non-transgenic under similar conditions (Figure 3b). Use of 200 mg l^−1^ 5-FC permitted rooting of 25% of the codA2 transgenic shoots. The 5-FC concentrations 100, 75, and 50 mg l^−1^ showed little or no marked difference in rooting inhibition between the transgenic and wild type tissues. Thus, we demonstrate that the *codA* gene can be used as an effective conditional negative selection marker in citrus cultivar Carrizo at a concentration of 5-FC of 100 mg l^−1^ for shoot regeneration while a concentration of 400 mg l^−1^ was necessary to inhibit rooting.

## Discussion

We have shown that *codA* is an effective negative selection marker. We produced and molecularly characterized transgenic citrus expressing one or more copies of the *codA* gene without any observable phenotypic abnormalities.

Furthermore, we have demonstrated that non-transgenic citrus internode tissue is sensitive to the presence of 5-FU (Table 1; Figure 2) and insensitive to 5-FC at concentrations below 200 mg l^−1^. In contrast, transgenic plants expressing the *codA* gene showed high sensitivity to 5-FC (Table 2). These results are consistent with previous publications using 5-FC as a selective agent in transgenic *L. japonicas* leaves (Stougaard 1993), *N. tabacum* leaves (Schlaman and Hooykaas 1997), *A. thaliana* suspension cells (Gallego et al. 1999), *H. vulgare* scutellar tissue (Koprek et al. 1999), and *B. napus* cotyledons (Babwah and Waddell 2000; Kobayashi et al. 1995; Stougaard 1993). We observed that 100 mg l^−1^ of 5-FC was sufficient to inhibit shoot regeneration from *codA* expressing transgenic tissue whereas non transgenic tissue was unaffected for regeneration at concentrations below 200 mg l^−1^. However, use of 5-FC for rooting inhibition required 400 mg l^−1^ for effective selection of *codA* transgenics.

In conclusion, this work demonstrates that the *codA* gene can be successfully used as a negative selectable marker in transgenic citrus, and is now available for use as a tool to facilitate precise genome modification strategies.
